# Prevalence of resistance to integrase strand-transfer inhibitors (INSTIs) among untreated HIV-1 infected patients in Morocco

**DOI:** 10.1186/s13104-018-3492-5

**Published:** 2018-06-08

**Authors:** Najwa Alaoui, Moulay Abdelaziz El Alaoui, Nadia Touil, Hicham El Annaz, Marouane Melloul, Reda Tagajdid, Naoufal Hjira, Mohamed Boui, El Mostapha El Fahime, Saad Mrani

**Affiliations:** 1 Faculty of Medicine and Pharmacy, University Mohammed V in Rabat, Av. Mohamed Belarbi El Alaoui, 6203 Rabat, Morocco; 2 Functional Genomic Platform, UATRS, Center for Scientific and Technical Research [CNRST], 10000 Rabat, Morocco; 3 Laboratory of Physiology, Genetics and Ethnopharmacology, Faculty of Sciences of Oujda, University Mohammed Premier, 60000 Oujda, Morocco; 4 Department of Dermatology and Venereology, Faculty of Medicine and Pharmacy, University Mohammed V in Rabat, Av. Mohamed Belarbi El Alaoui, 6203 Rabat, Morocco

**Keywords:** HIV-1, Integrase, Resistance mutations, INSTIs, Naïve patients, Morocco

## Abstract

**Objective:**

The integrase strand-transfer inhibitors (INSTIs) are an important class in the arsenal of antiretroviral drugs designed to block the integration of HIV-1 cDNA into the host DNA through the inhibition of DNA strand transfer. In this study for the first time in Morocco, the complete HIV-1 integrase gene was analysed from newly diagnosed patients to evaluate the prevalence of natural polymorphisms and INSTIs resistance-associated mutations in the integrase gene.

**Results:**

The 864pb IN coding region was successfully sequenced from plasma sample for 77 among 80 antiretroviral naïve patients. The sequences were interpreted for drug resistance according to the Stanford algorithm. Sixty samples were HIV-1 subtype B (78%), fourteen CRF02_AG (18%), two subtype C and one subtype A. Overall 81 of 288 (28%) amino acid IN positions presented at least one polymorphism each. We found 18 (36.73%), 42 (25.76%) and 21 (27.27%) of polymorphic residues assigned to the N-Terminal Domain, Catalytic Core Domaine and the C-Terminal Domain positions respectively. Primary INSTIs resistance mutation were absent, however secondary mutations L74IM, T97A were detected in four samples (5.2%). These results demonstrate that untreated HIV-1 infected Moroccans will be susceptible to INSTIs.

**Electronic supplementary material:**

The online version of this article (10.1186/s13104-018-3492-5) contains supplementary material, which is available to authorized users.

## Introduction

Since the introduction of combination therapy (highly active antiretroviral therapy, HAART) with protease inhibitors (PIs), nucleoside reverse transcriptase inhibitors (NRTIs) and non-nucleoside reverse transcriptase inhibitors (NNRTIs) in Morocco in 1998, the mortality and morbidity of HIV/AIDS patients has reduced [1]. These drugs suppress viral replication and reduce HIV viral RNA loads in the plasma of patients, thus helping to maintain the immune system, but they do not prevent escape through the emergence of drug resistant viruses and subsequent treatment failure.

According to previous Moroccan studies, the prevalence of resistance to NRTIs, NNRTIs and PIs are continuously increasing among drug-naïve and treatment experienced patients [2–4], thus, developing new drugs for AIDS treatment would be needed.

INSTIs are the latest antiretroviral (ARV) drugs class developed for the treatment of HIV-1 infections via the inhibition of DNA strand transfer [5]. To date three INSTIs, raltegravir (RAL), elvitegravir (EVG) and dolutegravir (DTG) are approved for clinical use [6–8]. They are potent ARV drugs offering more treatment options in naïve patients as well in pretreated patients with preexisting drug resistance or treatment complication [9–15]. Therefore, they have become an essential component of HAART used in many countries. Morocco has installed national programs following World Health Organization ARV guidelines and newer ARV salvage regimens including third-line drugs such as INSTIs will been introduced in the upcoming years. The aim of this study is to analyze, for the first time in Morocco, integrase (IN) sequence variability among ARV treatment naïve patients to determine the frequency of resistance mutations and the prevalence of natural polymorphisms of the IN gene and in order to estimate INSTIs efficacy prior to their introduction into the country.

## Main text

### Methods

#### Study population and samples

Plasma samples were collected for genotypic assay of the IN gene region from eighty seven HIV-infected, antiretroviral therapy-naïve patients originating from different geographic parts of the country enrolling at the dermatology department of Mohammed V Military Teaching Hospital in Rabat between the years 2009 and 2015. Demographic, clinical and laboratory data were collected for all patients. The quantitative HIV-RNA tests were performed using Cobas TaqMan HIV-1 Test, version 1.0 (Roche Diagnostics Systems, Germany, *P/N: 03542998 190*). CD4 cell enumerations were performed using the FacsCount instrumentation. (FacsCount, Becton–Dickinson, *P/N: 339010*). After viral load testing,eighty samples were used for sequencing assay. The detection of IN mutations by sequencing was unsuccessful for three samples.

The study was approved *by the Ethical Committee of Biological Research, Faculty of Medicine and Pharmacy–Rabat*, and was conducted with respect to legal aspects. Written informed consent was obtained from all participants before any data analysis procedure.

#### Genetic analysis and drugs resistance

HIV RNA was extracted from plasma using High Pure Viral RNA Kit (Roche Diagnostics Systems, Germany, *P/N: 11858882001*) and the integrase coding region (867 bp) was amplified by one-step reverse transcriptase polymerase chain reaction (RT-PCR) using MyTaq One-Step RT-PCR kit (Bioline, London, UK, *P/N: BIO-65049*) and the primer set KVL068 -KVL069 [16]. The Nested-PCR assay was carried out using MyFi DNA Polymerase kit (Bioline, London, UK, *P/N: BIO-21118*) and the primer set KVL070 and KVL084 [16]. Sequencing reaction was performed using BigDye Terminator v3.1 Ready Reaction Cycle Sequencing Kit (*P/N: 4337455*) with an ABI PRISM 3130XL Genetic Analyzer (Applied Biosystems) using the POP-7 polymer (*P/N: 4393708*). Data were analyzed by sequencing Analysis Software version 5.3.1 (Applied Biosystems, *P/N: 4360967*).

IN Sequences were assembled and aligned using DNA Dragon Sequence Assembler version 1.6.0 (Sequentix-Digital DNA Processing, Germany) and Muscle method in MEGA 6 software [17], respectively. All sequences were submitted to GenBank and registered under accession numbers: *KU609274–KU609350*.

**Fig. 1:**
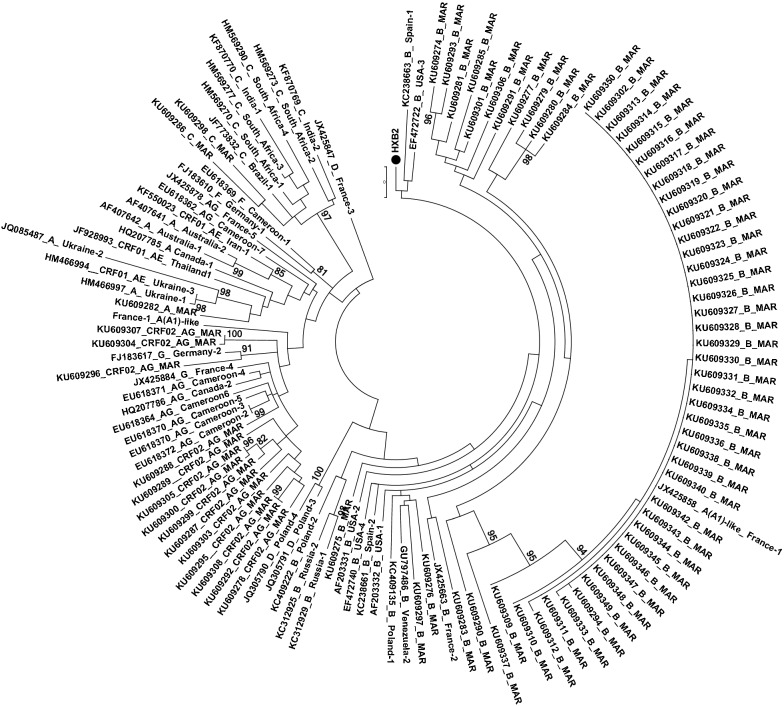
Molecular phylogenetic analysis by maximum likelihood method. Maximum likelihood phylogenetic tree based on nucleotide full sequences of HIV-1 integrase gene. Moroccan strains are indicated by accession numbers with the corresponding subtypes (KU609274–KU609350). Reference sequence HXB2 is marked with black circle. Representative sequences of different subtypes and CRFs are indicated by country name with the corresponding subtype. Bootstrap values (1000 replicates) less than 70% are not shown

HIV-1 subtyping and screening of IN polymorphisms in comparison with the HxB2 HIV-1 clade B consensus sequence (GenBank accession number K03455.1) were done using geno2pheno subtyping tools [18]. Phylogenetic tree was constructed by using Maximum Likelihood method, and Bootstrap resampling was performed 1.000 times for all sequences with MEGA 6 software (Fig. 1). INSTIs resistance mutations in IN sequences were interpreted using the Stanford HIVdb Program (Version September 23,2016) and screened for the presence of additional changes in 17 positions (V72I, T112I, S119PRTG, T124A, T125K, A128T, Q146K, M154I, K156N, V165I, V201I, I203M,T206S, S230N, D232N, V249I and C280Y) previously related to INIs resistance in vitro and frequently reported by different studies [19–23]. The variability at the D_64_D_116_E_152_ and H_12_H_16_C_40_C_43_ motifs, the residues that interact with the human lens epithelium derived growth factor (LEDGF/P75) and the degree of variability of the three functional areas of IN were also investigated.

### Results

Out of eighty patients, sixty-two (77.5%) were male. Median age was 36 years old, where patients with an age between 25 and 44 years old represent 61%. Sixty-five (81.25%) patients were suspected to have acquired HIV infection through heterosexual contact, four (5%) perinatally, and the mode of infection was unknown for 11 patients. The median of CD4+ T cell count and viral load at the time of sequencing for the available values were 409 cells/mm^3^ and 95,800 copies/ml respectively (Table 1).

**Table 1:** Demographic characteristics of HIV-infected drug-naïve Moroccan patients at the time of the sampling (during the period 2009–2015)

Characteristic	Number (80)	Frequency %	Descriptive statistic									
			Mean	s2	SD	Med	CV (%)	SE	Q1	Q3	IQR	Range
Sex			40.00	484.00	22.00	40.00	0.550	22.00	29.00	51.00	22.00	44.00
Men	62	77.5										
Women	18	22.5										
Age category (years)												
< 11	4	5	37.075	166.019	12.88	36	0.348	1.45	31	45.25	14.25	85
11–24	4	5										
25–44	49	61.25										
> 44	23	28.75										
Transmission route												
Heterosexual contact	65	81.25	20.00	340.50	18.45	13.50	0.923	10.654	4.00	29.500	25.50	45.00
Mother to child	4	5										
Not determined	11	13.75										
CDC category at HIV diagnosis												
Class A	28	35	20.00	31.50	5.612	19.50	0.281	3.240	16.00	23.500	7.50	15.00
Class B	13	16.25										
Class C	17	21.25										
Not determined	22	27.5										
CD4 cell count category (cells/ml)												
0–49	3	3.75	388.59	32,570.27	180.47	409	0.464	23.107	285.25	506	220.75	780
50–99	4	5										
100–199	6	7.5										
200–349	8	10										
≥ 350	41	51.25										
Not determined	18	22.5										
Viral load category (copie/ml)												
< 249	2	2.5	3.8E+05	7.9E+11	8.9E+05	9.6E+04	2.3E+00	1.1E+05	2.9E+04	2.6E+05	2.3E+05	5.6E+06
250–4999	5	6.25										
5000–29,999	10	12.5										
30,000–99,999	15	18.75										
100,000–999,000	25	31.25										
≥ 1000,000	7	8.75										
Not determined	16	20										

*S*^*2*^ variance, *SD* standard deviation, *SE* standard error, *Q1* superior quartile, *Q3* inferior quartile, *IQR* interquartile range, *Med* Median, *CDC* centers for disease control

**Fig. 2:**
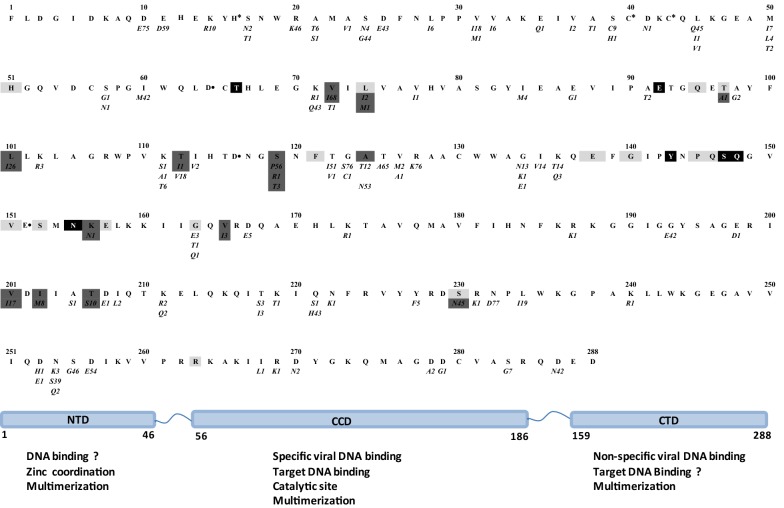
Frequency of amino acid sequence polymorphisms in the integrase gene with HIV-1HXB2 as the reference. Numbers below each position are the numbers of isolates with that specific polymorphism. Dark shaded boxes with white letters signify positions associated with primary resistance and lighter gray shaded boxes with black letters signify positions associated with secondary resistance as listed in the Stanford HIV Drug Resistance Database stated in September 2016. Dark gray boxes with black letters represent aa substitutions associated with INI resistance in vitro that occurred as natural polymorphisms [16, 20] found in our study. The N-terminal domain (NTD) is represented by AA acids at positions 1-50, the central core domain (CCD) at positions 51–212 and the C-terminal domain (CTD) at positions 213-288. The HHCC zinc-binding motif residues in the NTD are indicated by “*” and the DDE motif residues in the CCD are indicated by “•”

In 3 samples the sequencing failed and subtypes were unavailable. The final dataset for the baseline IN resistance included 77 individuals. IN sequencing was consistently successful at HIV viral loads higher than 66 copies/ml. Sixty (77.92%) were of HIV-1 subtype B, fourteen (18.18%) CRF02_AG, two (2.6%) subtype C and one (1.3%) subtype A. These data are also summarised in the IN phylogenetic tree shown in Fig. 1. The screening of sequences revealed that Overall 81 of 288 (28%) amino acid IN positions presented at least one polymorphism each. we found 18 (36.73%), 42 (25.76%) and 21 (27.27%) of polymorphic residues assigned to the N-Terminal Domain, Catalytic Core Domaine and the C-Terminal Domain positions respectively (Fig. 2). As expected, no polymorphism was found in the HHCC Zn^+^-binding motif and in the catalytic triad DDE. Also the amino acid in IN positions that have been identified as critical for interaction with LEDGF/P75, H12, L102, A128, A 129, C130, W131, W132, I161, R166, Q168, E170, H171, T174, M178 and Q 214 [24–26] were conserved. The substitutions detected in more than 95% of samples were D10E, G123S, R127K and N232D, none of which is ascribed to INI resistance. None of the primary amino acid mutations in IN positions 66, 92, 140, 143, 147, 148 and 155 listed in the Stanford resistance algorithm were found in this study, while secondary mutations L74IM and T97A associated with drug resistance to RAL and EVG were observed only in four patients (5.2%) (Additional file 1). The L74I was observed in two strains, a subtype B (KU609297) and a subtype A (KU609282). The L74M was observed in one subtype B strain (KU609337) and T97A was observed in one CRF02_AG strain (KU609303). No mutation associated with DTG resistance was observed among all studied patients. Other mutations in IN positions not included in Stanford list but frequently reported in regards to INIs resistance in vitro V72I, T112I, S119PRT, T124A, K156N, V165I, V201I, I203M,T206S and S230N have been detected with different frequencies (Fig. 2). Finally there were no significant associations (p > 0.05) between resistance mutations with exposure category, viral load, Transmission route and CD4 T cell count.

### Discussion

Three INSTIs, RAL, EVG and DTG have been approved for clinical use by the FDA and European Medicines Agency [6–8]. These compounds have proven to be highly efficient for the treatment of both ARV-naïve and -experienced individuals even with preexisting drug resistance or treatment complication [9–15], thus INIs has rapidly became an important class in the arsenal of ARV drugs. Eighty HIV-1 untreated patients were recruited in this study in order to examine the variability within the IN gene at positions associated with resistance to INIs. Despite the limited size of the study population, these results are in good agreement with the current situation of HIV in Morocco as reported in the national report on AIDS [1]. The high prevalence of subtype B among infected Moroccan population has been previously reported by other studies which suggested that it was due to the presence of a close relationship between Morocco and European countries [3, 4]. Detailed phylogenetic analysis of IN sequences showed that all CRF02_AG isolates were related to strains found in central Africa and Europe, which agreed with previous reports that suggested that increasing prevalence of CRF02_AG might be associated with increasing immigration from sub-Saharan Africa to Europe via Morocco [27, 28]. Of the 288 IN amino acids positions, 81(28.12%) had one or more variants. This rate (28%) is lower to that reported by Rhee et al. (39.9%) in different subtypes of group M integrase sequences obtained from more than 1500 individuals who were INI-naïve, and either ARV-naïve or ARV-experienced [29]. This digit indicates the relative conservation of the protein in untreated Moroccan’s patients. The analysis showed conservation of the HHCC Zn^+^-binding motif, the catalytic triad DDE, and several important IN residues involved in the chemical bond and hydrophobic contact with LEDGF/P75; which an essential HIV integration cofactor linking IN to chromatin [24–26]. The conservation of these specific structural domains is strictly necessary for the correct performance of HIV-1 IN functions [30].

Importantly the major resistance mutations with reduced susceptibility to RAL, EVG and DTG were totally absent. The absence of such mutations in our study is consistent with the results of several studies in treatment-naïve patients [19, 20, 31–35] and with the fact that the transmission of the drug resistance is unlikely in populations previously unexposed to INIs treatment [36]. Only three secondary drug resistance mutations included in Stanford list were observed in 4 strains. These mutations have been previously been described as polymorphic, occurring in 1–2% of IN sequences, observed in subtypes A, B, C, D, CRF01_AE and CRF02_AG [29]. These mutations contribute to INI resistance only in the presence of primary INI resistance mutations [19, 37]. Moreover, there were three amino acid substitutions of unknown significance at position 163 that were encountered in subtype B and CRF02-A/G strains: G163E, G163T and G163Q. In IN residues, it is usually considered non polymorphic in all subtypes except subtype F [38]. Regarding DTG R263K resistance mutation, no strain from our study exhibited this mutation, whereas the L101I and T124A mutations were found in 26 and 12 strains, respectively. These mutations were previously shown to be selected in vitro in the presence of DTG and have shown little impact on virological response to DTG [39, 40]. Conversely, specific additional mutations in amino acid IN positions 72, 112, 119,156, 165, 201, 203, 206, and 230 occurred with different prevalence in subtype B and non-B HIV-1 variant were more common. These mutations have not been described to be associated with RAL or EGV resistance [20, 21]. In the same way, it has been reported that in the absence of primary mutation, all these secondary mutations had little if any effect on drug susceptibility in vitro, thus suggesting rather a secondary role for viral fitness rescue and/or increasing resistance [19]. Furthermore many previous genotypic studies on HIV-1 IN in treatment-naïve patients living with various viral subtypes in different countries: England, Spain, South Africa, Sub-Saharan Countries, Thailand, Indonesia and Korea have showed that these differences are natural polymorphisms [32–34, 41–43]. According to previous studies, and the fact that INIs have not yet been introduced in Morocco, all secondary and additional mutations identified in this study are also likely natural polymorphisms.

In conclusion, these results demonstrate that untreated HIV-1 infected Moroccans are likely to benefit from INSTI-based drug regimens, particularly given the rising issues related to drug resistance against reverse transcriptase inhibitors that are currently used in Morocco.

## Limitations

The authors wish to highlight that our limitation consist on sample size, more participants are necessary before a large introduction of integrase inhibitors into our country.

## Additional file

**Additional file 1.** Distribution of IN mutations in subtypes B and non-B in therapy-naïve patients. Secondary and additional mutations screened in 17 positions (V72I, T112I, S119PRTG, T124A, T125K, A128T, Q146K, M154I, K156N, V165I, V201I, I203M, T206S, S230N, D232N, V249I and C280Y) using the Stanford HIV Drug Resistance Program (Version September 23, 2016), all mutations identified in this study are likely natural polymorphisms.

## Abbreviations

INSTI: integrase strand-transfer inhibitor
IN: integrase
INI: integrase inhibitor
CCD: catalytic core domain
HAART: highly active antiretroviral therapy
ARV: antiretroviral
PI: protease inhibitor
NRTIs: nucleoside reverse transcriptase inhibitors
NNRTI: non-nucleoside reverse transcriptase inhibitors
RT-PCR: reverse transcriptase polymerase chain reaction
RAL: raltegravir
EVG: eviltegravir
DTG: dolutegravir
