# Supplementary material for: Prevalence of resistance to integrase strand-transfer inhibitors (INSTIs) among untreated HIV-1 infected patients in Morocco
Source: BMC Res Notes. 2018 Jun 8;11:369. doi: 10.1186/s13104-018-3492-5 (PMC5994051; doi:10.1186/s13104-018-3492-5)
Supplement: Supplementary file 1 — Additional file 1. Distribution of IN mutations in subtypes B and non-B in therapy-naïve patients. Secondary and additional mutations screened in 17 positions (V72I, T112I, S119PRTG, T124A, T125K, A128T, Q146K, M154I, K156N, V165I, V201I, I203M, T206S, S230N, D232N, V249I and C280Y) using the Stanford HIV Drug Resistance Program (Version September 23, 2016), all mutations identified in this study are likely natural polymorphisms. [file 13104_2018_3492_MOESM1_ESM.doc]

| Mutation | Subtype B(N= 60) | Non-cladeB(N= 17) | Total (N=77) |
| --- | --- | --- | --- |
|  | n (%) | n (%) | n (%) |
| V72I | 56(93.34) | 12(85.71) | 68(88.31) |
| L74I^a^ | 1(1.67) | 1(5.88) | 2(2.59) |
| L74M^a^ | 1(1.67) | 0 (0) | 1(1.3) |
| T97A^a^ | 0(0) | 1 (5.88) | 1(1.3) |
| L101I | 11 (18.34) | 15(88.24) | 26 (33.76) |
| T112I | 1(1.67) | 0(0) | 1(1.3) |
| S119P | 54 (90) | 2(11.76) | 56(72.72) |
| S119T | 3(5) | 0(0) | 3(3.89) |
| S119R | 1(1.67) | 0(0) | 1(1.3) |
| T124A | 5 (8.33) | 7 (41.17) | 12(15.6) |
| K156N | 1(1.67) | 0(0) | 1(1.3) |
| V165I | 3(5) | 0(0) | 3(3.89) |
| V201I | 5(8.34) | 12(70.58) | 17(22) |
| I203M | 1(1.67) | 7(41.17) | 8(10.38) |
| T206S | 5(8.34) | 5(29.41) | 10(13) |
| S230N | 45(75) | 0(0) | 45(58.44) |

a : Secondary mutations reported by the Stanford HIV Drug Resistance Database updated on September 23, 2016
